# Everyday Challenges Faced by Polish Teenagers During the COVID-19 Pandemic in the Context of Selected Demographic Factors

**DOI:** 10.34763/jmotherandchild.20212503SI.d-21-00026

**Published:** 2022-02-09

**Authors:** Magdalena Korzycka, Martyna Bójko, Katarzyna Radiukiewicz, Anna Dzielska, Anna Oblacińska, Anna Fijałkowska

**Affiliations:** 1 Department of Children and Adolescent Health, Institute of Mother and Child, Warsaw, Poland; 2 Department of Cardiology, Institute of Mother and Child, Warsaw, Poland

**Keywords:** Pandemic, coronavirus, isolation, adolescents, school, family

## Abstract

**Background:**

The enforced restrictions, including physical isolation and school lockdowns after the outbreak of the COVID-19 pandemic, have brought about anxiety and uncertainty the younger generation.

**Objective:**

The main objective is to analyse the everyday challenges faced by adolescents in Poland during the time of social isolation in the COVID-19 pandemic.

**Material and methods:**

A nationwide, online survey of adolescents aged 11–18 (N=2408) was conducted in April 2020. Quantitative and qualitative analyses were undertaken. Differences in the everyday challenges experienced during the COVID-19 pandemic connected with gender, age and place of residence were analysed.

**Results:**

Girls were significantly more likely than boys to perceive the limitations in contacts with others (friends and family) and the concerns about the health of relatives as a big difficulty. Among the youngest students (11–12 years of age), the lack of contact with friends and family and worries about their health and the fear of infection ranked higher than for other students. For the oldest (17–18) the lack of private time and space and not being able to meet one's boyfriend/ girlfriend were the most troublesome. The necessity to stay at home and the inconvenience resulting from the lack of outdoor exercise were ranked higher by urban students than by students living in rural areas.

**Conclusions:**

When planning campaigns in the near future to support the mental health of adolescents in the context of the pandemic it is recommended to include especially the youngest adolescents and those living in small and medium-sized cities.

## Introduction

The outbreak of the COVID-19 pandemic has affected the lives of people across the globe, not only with regards to health – it has also caused turmoil in daily life, including changes in the form of work, study and contact with other people. [1] The introduced restrictions, including physical isolation, aimed at containing the spread of the pandemic have posed a huge challenge both for individuals and families, as well as the society as a whole. [2] The imposed isolation has made it necessary to stay indoors at all times, and – as a result of the epidemiological threat – concerns about the health and safety of the loved ones have increased. New rules concerning everyday life have brought about anxiety and uncertainty both among adults and the younger generation. [3,4] Due to the enforced restrictions – including physical isolation and school lockdowns – students have experienced emotional problems, feelings of loneliness and even sleep disorders. [5,6,7] Considering health effects, the pandemic has mostly affected the elderly and those with coexisting conditions (including hypertension, lung diseases, diabetes): that is, those who generally experience SARS-CoV-2 infection more severely than younger people, and are more likely to require hospitalization. [8,9] Although younger people are less likely to be severely affected by the coronavirus, social isolation imposed by the restrictions intended to contain the transmission of the virus has increased the risk of the development of depression in this age group [10, 11, 12]. Adolescents, who at the beginning of the pandemic in Poland were subject to more restrictions than any other social group, also faced a difficult situation. Initially, adolescents under the age of 18 were not allowed to leave home without an adult [13], later the rules became more lenient, and only children under the age of 13 were affected. [14] In October 2020, the regulations were tightened again, prohibiting children under 16 from moving about without an adult between 8 am and 4 pm. [15] This ban was lifted temporarily for the winter holidays. [16] The constant presence of the family, the frequent lack of private space, the inability to leave the house on their own, the tense atmosphere among the household members related to the epidemic situation – all these factors greatly affected the well-being of children, thus reducing their comfort. [17]

Research/surveys on the lives of adolescents were conducted during the period of the greatest restrictions. [18,19]

The main objective of this research is to analyse the challenges faced by adolescents aged 11– 18 in Poland during the time of social isolation and the most stringent restrictions related to the COVID-19 pandemic, and the differences in the occurrence of these problems depending on gender, age and place of residence of the adolescents studied.

## Material and methods

### Organisation of the survey and persons surveyed

A nationwide, online survey of young people aged 11–18 was conducted between April 13–26, 2020. The coordinating center for the survey "Youth and COVID-19" was the Institute of Mother and Child (IMiD).

Two recruitment channels were employed:

- An invitation to the survey via e-mail – providing a link to the online survey to schools located throughout Poland by school nurses, youth counselors and teachers cooperating with the Institute of Mother and Child (IMiD);
- An announcement with an invitation to the study posted on the social networking sites of IMiD and the IMiD Foundation in Warsaw,

In all, 3351 completed questionnaires were received. A final sample of 2408 adolescents was obtained. The procedure of the study entitled ‘Youth and COVID-19’ received a positive opinion of the Bioethical Committee at the Institute of Mother and Child (opinion No 24/2020). Detailed information regarding the organisation of the study is presented in a separate report. [20]

## Research tools and indicators

The online survey questionnaire included five groups of questions to assess various areas related to children and adolescents' functioning during the isolation: 1. Ways of coping in the pandemic situation; 2. Everyday problems associated with the pandemic; 3. Learning problems; 4. Adolescents’ needs in the pandemic situation; and 5. How adolescents can help others during the pandemic. The discussed analyses included a set of closed questions that related to everyday problems experienced by young people during the COVID-19 pandemic, and responses to an open-ended question related to experiencing everyday problems. Demographic variables were also included in the analyses.

## Demographic variables

The age was divided into 3 categories: 11–12 years, 13–16 years, and 17–18 years.

The question on the place of residence, which reads: *Where do you live?*, had the following response options: *In the countryside, In a small city (up to 20,000 inhabitants), In a larger city (between 20,000 and 100,000 inhabitants), In a large city (more than 100,000 inhabitants)*. The variable was analysed in 3 categories by combining the middle two responses.

## Challenges experienced by young people during the pandemic

**Figure 1:**
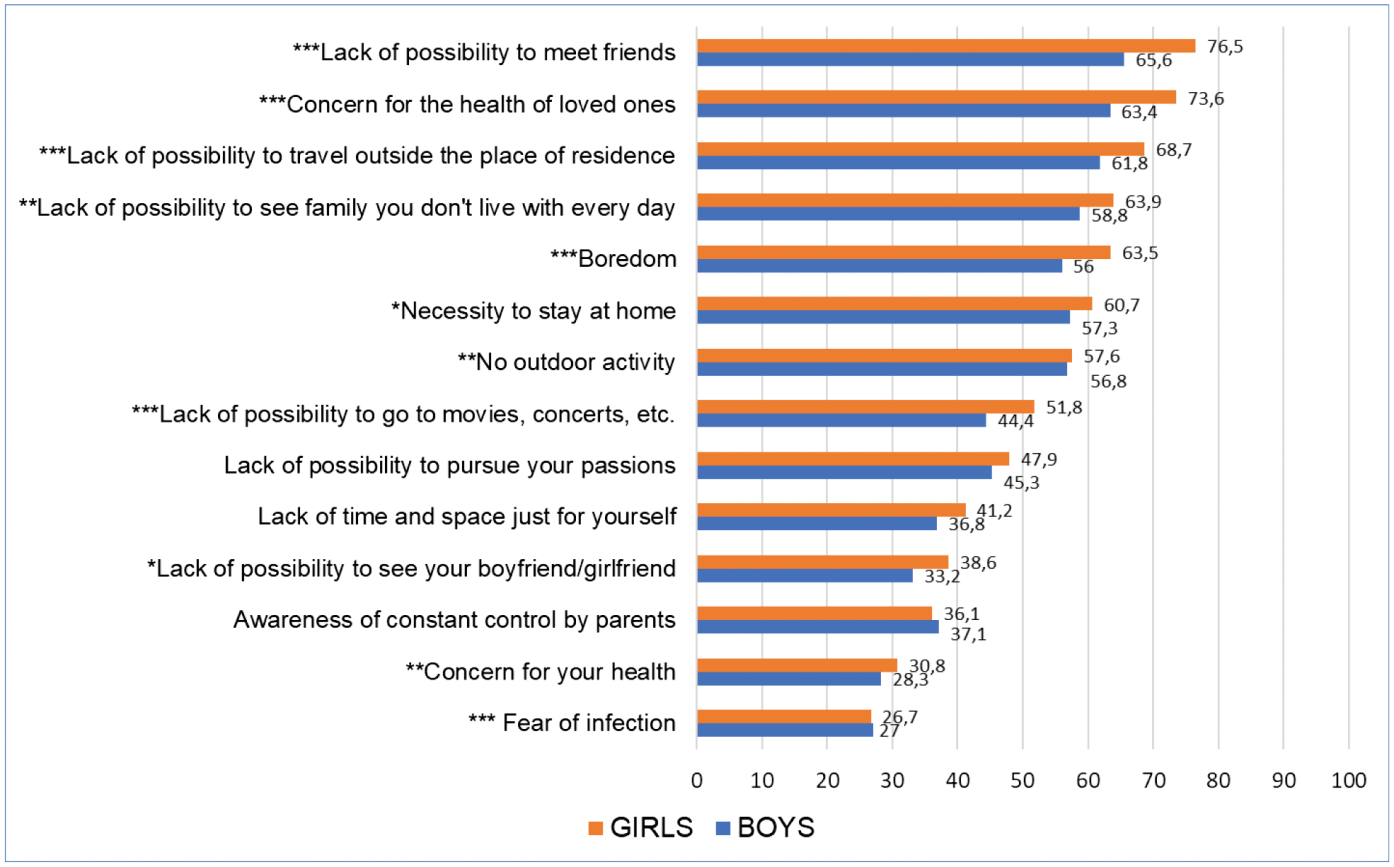
Ranking of problems experienced by youth during a pandemic, by gender (%); *p<0,05; **p<0,01; ***p<0,001;

**Figure 2:**
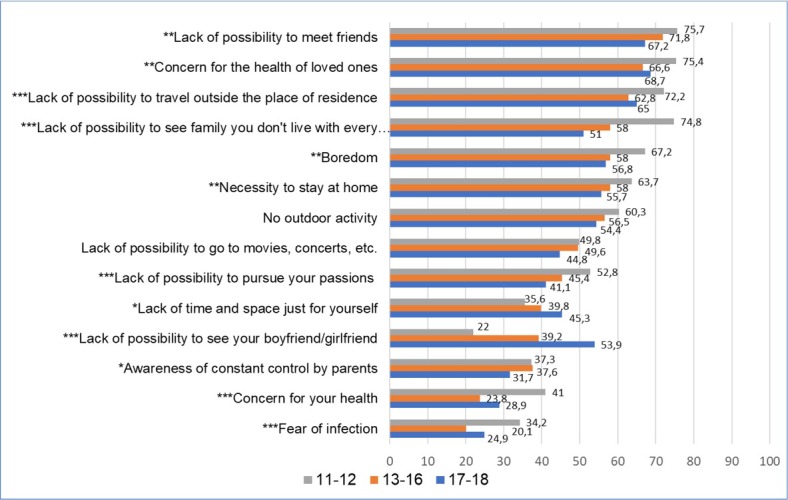
Ranking of problems experienced by youth during a pandemic, by age (%); *p<0,05; **p<0,01; ***p<0,001;

**Figure 3:**
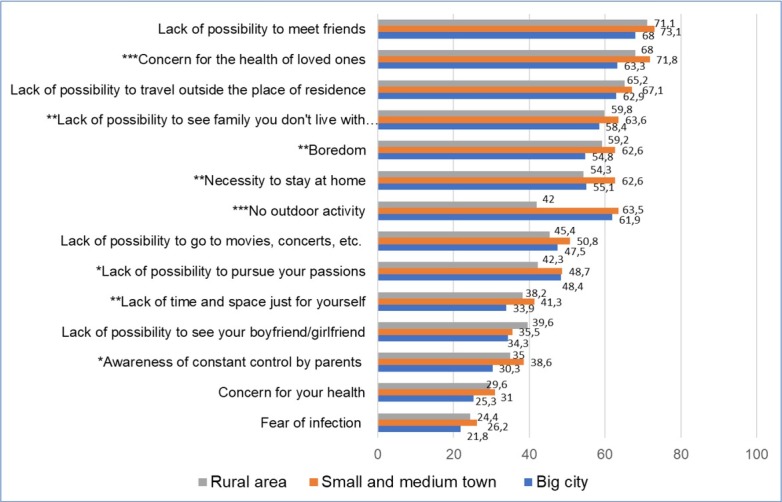
Ranking of problems experienced by youth during a pandemic, by place of residence (%); *p<0,05; **p<0,01; ***p<0,001

The full list of 14 statements regarding the daily problems associated with the COVID-19 pandemic is quoted in Table 1 and Figs 1–3.

Adolescents rated the above 14 areas of perceived difficulty related to the COVID-19 pandemic by answering the question: *How big a problem are the following situations, thoughts, and feelings related to the epidemic for you*, marking their opinion on a scale of 1 to 5, where 1 means *not a problem at all* and 5 means *a very big problem*. For the purpose of the analyses, the two extreme responses were combined. A ranking of problems was also created – from the lowest to the highest percentage of negative evaluation (big problem).

All additional statements in the category ‘Other’ provided by the young people regarding the challenges faced during the pandemic were organised and categorised according to the methodology of qualitative content analysis. [21] There were 154 additional opinions concerning problems that were not included in the set of closed questions. Based on these opinions, 10 new thematic areas were identified, and are presented in the results description section.

## Statistical analyses

Quantitative and qualitative analyses were undertaken. [22] Descriptive statistics were used in the statistical analysis of the quantitative data. The relationship between the assessment of each of the 14 statements on daily problems related to the prevailing COVID-19 pandemic and the gender, age and place of residence was tested using the chi-square test. Quantitative analyses were conducted using IBM SPSS v. 25 software. The value p<0.05 was accepted as the level of significance.

The preliminary interpretation of the statements and the development of a categorisation key, taking into account the most frequent thematic areas, preceded the categorisation and the principal analysis of the content obtained on the basis of the adolescents' own statements about additional problems.

## Results

### Study group

The majority of the surveyed group were girls (60.1%). The highest number of responses was obtained from adolescents aged 13–16 (52.5%), and the representatives of small and medium towns (59.1%).

### Problems experienced by adolescents during the pandemic

The situation that most often posed a great problem for adolescents during the pandemic, which approximately three-quarters of them complained about (72.2%), was the lack of possibilities to meet with friends (Table 1). Adolescents described their own fears about the health of the closest family as a big problem (69.6%), as was their the inability to travel outside of their place of residence (66%). Other situations which constituted a considerable problem for marginally more than half of the teenagers included: the inability to meet with family, boredom, and also the necessity to stay at home and lack of outdoor exercise (Table 1).

**Table 1:** Ranking of problems experienced by young people during the pandemic – in the total group in the order from most to least frequent (%)

Problems	Subjective assessment of the importance of the problem (N=2408)		
	Lack of problem	Medium problem	Big problem
Lack of possibility to meet friends	14.6	13.2	72.2
Concern for the health of loved ones	13.2	17.2	69.6
Lack of possibility to travel outside the place of residence	19.4	14.6	66.0
Lack of possibility to see family you don't live with every day (e.g. grandparents, cousins)	19.9	18.2	61.9
Boredom	22.7	16.7	60.6
Necessity to stay at home	25.0	15.7	59.3
No outdoor activity	27.9	14.8	57.3
Lack of possibility to go to movies, concerts, etc.	30.0	21.2	48.8
Lack of possibility to pursue your passions in extracurricular activities that cannot be arranged online	36.5	16.6	46.9
Lack of time and space just for yourself	42.5	18.0	39.5
Lack of possibility to see your boyfriend/girlfriend	57.4	6.1	36.5
Awareness of constant control by parents	44.9	18.6	36.5
Concern for your health	48.6	21.6	29.8
Fear of infection	52.9	22.0	25.1

## Ranking of problems experienced by adolescents during the pandemic according to demographic factors

Girls were more likely than boys to negatively assess the difficulties encountered (Fig. 1). Moreover, depending on gender, individual problems were of different importance. Girls were significantly more likely than boys to perceive the limitations in contacts with others, e.g. lack of opportunities to meet with friends and family, as a difficulty. Also, the concerns about the health of relatives more often constituted an issue for them. Conversely, the lack of opportunities to pursue hobbies or the awareness of being controlled ranked higher for boys.

The assessment of twelve out of fourteen analysed problems presented in the ranking differed considerably depending on *the age* and *place of residence* of the examined adolescents (Figs. 2, 3). In most cases, the youngest students (11–12 years of age) notably more often than the rest of the respondents assessed the situations and feelings related to the pandemic as a great problem. In this group, the greatest difficulties were associated with the lack of contact with friends and family. Worries about their health and the fear of infection also ranked higher than for other students. On the other hand, in contrast to other students, the lack of private time and space and not being able to meet one's boyfriend/girlfriend were the least troublesome for them.

The inconvenience resulting from the lack of outdoor exercise, as well as the lack of opportunities to develop one's passions, were ranked higher by urban students than by students living in rural areas (Fig. 3). On the other hand, the necessity to stay at home and the awareness of constant control by parents were the circumstances most frequently assessed as a problem by students from small and medium-sized cities.

## Additional difficulties related to the COVID-19 pandemic

The results of the analyses demonstrated that additional information concerning the problems experienced during the first weeks of the pandemic were more readily shared by girls than boys (N=104 vs. N=50).

The first issue most often mentioned by the teenagers was curricular overburdening (37 answers). Girls pointed to this most often, complaining that *Teachers send more assignments than we manage to do in normal lessons*. Some of the young people also pointed out that due to remote lessons they spend a lot of time working on the computer – *Because of online lessons I spend too much time at the computer; I sit too much at the laptop or phone, my eyes start to burn (…)*

Quite often young people also pointed out the lack of direct meetings with teachers and classmates (31 answers). *It is difficult to do lessons alone at home without the direct help of a teacher* – young people wrote, who also missed *laughing with their classmates side by side, sitting at one desk*. Slightly less frequently students stressed that they missed the way they functioned before the pandemic (27 answers), the ‘normal times’ – girls most often indicated this problem. They missed *the opportunity to live a normal life, everything changed for the worse in a split second*.

Both boys and girls also pointed to the lack of movement and physical activity (14 answers) during the lockdown. *Football trainings are a big problem because I love them* – we read in the responses. *At home I feel like I'm in prison, locked in, with no way out*. Many complained that they could not *go biking in the forest or walk the dog*.

Anxiety due to uncertain future (10 answers) was particularly noticeable in girls. *I am overwhelmed by the uncertainty connected with exams* – students wrote. They were also afraid of not being *able to fulfil their plans for this year (concerts of idols, camps, holidays abroad)*.

A noteworthy problem that was mainly pointed out by the girls was the lack of opportunity to meet with a psychologist (8 answers). *Adults think everything’s okay with us and don't give us psychological care, which I personally need because I'm not able to talk to anyone in my family about things like fear and panic during these times*, they wrote. Students also pointed to the *lack of access to specialist medical care* in general.

The information hype surrounding the coronavirus (8 answers) was also troublesome for everyday life. Young people were exasperated by the constant *deliberations about the pandemic. The panic spun by others* disturbed them.

An additional burdensome circumstance was the presence of family nearby, being forbidden to go out without adult supervision (7 answers)*. Not only were the adolescents unable to go for a walk or to the shop on their own*, but in many homes they were ‘doomed’ to *constant company of their younger siblings*. Technical problems (6 answers) were among the issues mentioned most often by the boys. The daily routine (6 answers) shrank to just a few activities – the youth complained *about doing the same activities over and over again, and lack of motivation, desire to do homework and study*.

## Discussion

The analyses conducted in the Institute of Mother and Child have confirmed that the restrictions introduced during the lockdown have posed a challenge for the youth. The most frequent issue indicated by the teenagers was the lack of possibility to meet with friends (72.2%). Adolescents, who naturally have a greater need for contact with peers than adults, have been affected by the social isolation caused by the pandemic particularly acutely [23]. This is confirmed by studies conducted in many countries during the lockdown [24, 25, 26]. As studies indicate, the lack of direct interaction with a peer group has a negative impact on the well-being of children and adolescents. [27] During the first months of the pandemic, in addition to the lack of meetings with friends, adolescents also suffered from the fear for their own life and health and that of their loved ones, the symptoms of anxiety and even depression were more frequent, which is also confirmed by foreign studies. [5] These symptoms did not affect only adolescents, but also younger children, who much more often than before the pandemic experienced stress, sadness and anxiety. [28]

The presented research carried out by IMiD has confirmed that for a large percentage of adolescents the fear for the health of their close relatives constituted a serious issue (69.6%). Such fear was considerably more often experienced by girls and the youngest students. Teenagers were rarely troubled by the state of their own health. Similar results were obtained in the studies conducted during the isolation among adolescents from Cracow (Poland) and surrounding areas. [29]

The low concern about their own health may result from the awareness of the respondents that adolescents are much less at risk of severe coronavirus-induced disease and hospitalisation than the elderly, which is confirmed by studies. [9] However, the younger generation is also at risk of infection and a severe or long duration of the disease, more than seemed to be the case in the initial weeks of the pandemic, although there are still important gaps in the identification of the various clinical consequences and risk factors for severe disease among children and adolescents. [30] Despite a lower risk of severe disease, compared with adults, an increase in the number of cases and the occurrence of various health complications was also reported among the youngest generation during the pandemic. [31]

According to the American Academy of Pediatrics (AAP), a relatively small but growing percentage of children and adolescents experience post-COVID or long-term physical and psychological consequences of SARS-CoV-2 virus infection. [32] These include, among others in addition to the long-lasting symptoms experienced during the acute phase, post-viral symptoms such as chronic fatigue syndrome, pulmonary and cardiovascular complications, and cognitive difficulties or mood changes. [33] A study conducted in Italy until November 2020 reported that more than half of children aged 6 to 16 years who got COVID-19 had at least one symptom lasting longer than 120 days, and among UK adolescents aged 12 to 16 years, 14.5% of patients were still symptomatic five weeks after their first infection. [34] In the context of the low assessment of the problem in the form of fear and anxiety about one's own health among adolescents, it is also worth mentioning after Długosz P. (2020) about the so-called psychological phenomenon of unrealistic optimism. [35] People often believe that unpleasant events will not affect them personally but pose a threat to other people around them. On the other hand, in the study conducted among the adolescents from Cracow and surrounding areas, the level of fear of infection increased with growing interest in the pandemic and the risk posed by the coronavirus. [29] In the study described, coordinated by IMiD, the adolescents who expressed their additional opinions complained about the flood of information concerning the coronavirus. Exposure to a large amount of information on the pandemic may reduce the sense of security, and even give rise to uncertainty and fear in young people. Therefore there is a need to properly educate and inform young people about the risk, but in a reliable way that does not induce fear, but makes them aware of how to protect themselves and loved ones. An example of such an initiative aimed at educating young people during lockdown is the information campaign for teenagers using graphic messages. [36] It was part of the survey ‘Youth and COVID-19’, discussed in this article. Infographics aimed at young people were part of this campaign and they were made available on social media, on the IMiD website and communicated to young people through schools. The infographics included opportunities for physical activity and hobbies during home isolation, occupational hygiene, hygiene during pandemics (masks, keeping physical distance), as well as healthy eating and safe use of electronic media.

For as many as 2/3 of the respondents the lack of possibility to travel outside the place of residence constituted a serious issue. Similar conclusions concerning the limitations with regards to social gatherings and mobility were drawn on the basis of another Polish qualitative-quantitative study, also conducted at the very beginning of the pandemic. [37] Other studies on psychosocial reactions of children and their parents to health crises indicated that isolation aimed at containing a disease is a traumatic experience for both children and parents. In some individuals it even leads to symptoms characteristic of post-traumatic stress disorder. [38] It is estimated that girls are twice as likely to experience post-traumatic stress disorder as boys. [39] It should be noted that both in our analysis and in other studies, the level of fear and anxiety associated with the pandemic, as well as the frequency of experiencing difficulties during this period, was higher in the population of girls than in boys. [40, 41]

Our study has revealed that the evaluation of the problems arising from the pandemic is dependent also on age. Adolescents between the ages of 11 and 12 were considerably more likely to evaluate the presented problems as serious. It should be emphasised that, while the youngest adolescents viewed – for instance – limited contacts with family and peers in this way, the teenagers from the oldest age group notably more often considered the lack of opportunities to meet with boyfriends/girlfriends as a great difficulty. During childhood and early adolescence, relationships with family and friends play a key role. [42] At the threshold of adulthood, 17–18-year-old adolescents more often than younger teenagers establish close emotional relationships and their relationship with their parents becomes of secondary importance. According to research, over one-third of youth aged 13 and almost three-quarters of 17-year-olds have experienced an important romantic relationship. [43] Young people in this phase of life also have an increased need for autonomy and independence, and a related need to make time and space just for themselves. [42]

In the light of our analyses, noteworthy differences have been observed in the assessment of the problems experienced by adolescents during the pandemic, to the disadvantage of the students residing in urban areas, especially small and medium-sized cities. In cities adolescents were more likely to view the need to stay at home, boredom or the lack of outdoor exercise as a problem than the adolescents residing in rural areas. When interpreting the differences dependent on the place of residence, it is important to consider the characteristics of rural and urban areas that may have been vital in the evaluation of the difficulties experienced by the young people. Rural environments are characterised by stronger social ties, development of cooperation, as well as a sense of community and belonging. [44]

The results obtained from the assessment of the difficulties experienced during the pandemic in terms of demographic characteristics also confirm the nature of small and medium-sized cities. These cities are the subject of national and international analyses and reports. [45, 46] Many of them need support, including new ideas for development; this is of particular importance also due to the epidemiological situation, which in 2020 exacerbated problems on the labour market, and caused deterioration of the living conditions of the inhabitants. [47]

Considering the differences in health concerns based on the place of residence, a study conducted during the isolation in China provided different results. [48] Among students, urban residence was a protective factor against anxiety arising from the coronavirus pandemic. [49]

An additional problem most often cited by the youth in the open-ended question was curricular overburdening. Students wrote about being overloaded with both online lessons and an overabundance of school assignments to complete individually. They also felt acutely the lack of meetings with teachers and class peers, which proves that remote learning cannot fully replace the traditional form of education based on meeting in person at school. [25] When the main channel for contacting the world is through electronic media, the technical problems may also constitute quite a challenge. [20] School is also a place where students can get the support they need from peers, teachers, school counsellors and psychologists. [50]

Teenage years are often the time of difficult relations with parents. [51] It is not a surprise, therefore, that the opinions expressed by the adolescents indicated a problem arising from the necessity to spend time with the closest family, including younger siblings, and frequent conflicts at home. According to the study, almost one in eight adolescents in Poland has complained about the decline in relations with household members, and one in six admitted to more frequent quarrels with family members. [52] During the pandemic, the future became the great unknown for young people, which gave rise to anxiety. This uncertainty of not knowing when everything would return to ‘normal’ is also pointed out by other researchers. [53] In the light of Polish studies, more than half of adolescents had difficulty in falling asleep, in every sixth adolescent an increase in depressive mood was observed, while every fifth adolescent had psychosomatic problems (abdominal pain, headaches, difficulty in falling asleep, nervousness, depression or bad mood, lack of energy). [52, 54] In view of all these intense fears and psychosomatic complaints among children and adolescents related to the pandemic, the problem of providing continuity of psychological and psychiatric care for adolescents during this difficult time, which is also pointed out by researchers, is particularly worrying. [4] According to experts, the effects of the pandemic on mental health may continue to be felt long after it has ended. [55] In this context, it is important to rapidly diagnose students' problems, using the possibilities offered by telemedicine also in the framework of psychiatric and psychological therapy, as well as to undertake inter-institutional interventions [52] and to develop and implement prevention programmes aimed at supporting young people emotionally. An example is the project entitled ‘zatroskani.pl – zdrowie psychiczne dzieci i młodzieży w Polsce [concerned.pl – mental health of children and young people in Poland]’, implemented by the Foundation for Health Education and Psychotherapy, which aims to help children and young people in the area of mental health. [56] This project focuses on the improvement of mental health of children and adolescents mainly in the context of the problem of abuse and addiction to digital media, which, due to isolation and remote learning, increased significantly during the COVID-19 pandemic. Various public and non-governmental institutions in Poland launched toll-free crisis support helplines during the pandemic, also for children and adolescents. An example of this is the nationwide psychological support telephone hotline aimed at people struggling with mental health problems and psychological difficulties related to the current epidemiological situation and its consequences, which was launched by the Mokotow Mental Health Center of the Institute of Psychiatry and Neurology: 222 990 431. On the other hand, an example of a non-governmental initiative is the 24-hour helpline for children and adolescents, run from 2019 by the ITAKA Foundation: 800 080 222 and the Youth Helpline: 22 484 88 04. A helpline for children and young has been run for more than 10 years by the Empowering Children Foundation: 116 111.

### Limitations and strengths of the study

Taking into account the process of collecting data, the studied sample is not representative. While conducting the analyses, the researchers were also aware of the disproportion in the size of the analysed sub-groups (the relatively small size of the group of the oldest teenagers, aged 17–18). One of the advantages of the presented research is the inclusion of both quantitative and qualitative analyses (mixed methods), as well as the inclusion of demographic variables. The additional information on the daily difficulties experienced by adolescents during isolation is a valuable addition to the categories of problems proposed by the researchers. They illustrate well the difficult situation faced by adolescents during the pandemic.

## Conclusions

1. Teenage girls experienced increased difficulties during the pandemic more often than boys.
2. In the light of the presented results, the greatest emotional costs caused by the pandemic were borne by the youngest participants of the study: 11–12-year-olds, who significantly more often than others assessed that the situations and feelings associated with the pandemic were a serious problem for them. Therefore, when planning campaigns in the near future to support the health of adolescents – also their emotional functioning in the context of the pandemic – it is recommended to include the youngest adolescents.
3. The problems arising from isolation during the first weeks of the pandemic were most acutely felt by young people from small and medium-sized cities. Therefore, it is worthwhile to pay special attention to this group when planning further in-depth research and when building comprehensive strategies aimed at supporting young people in the context of broadly defined health.
